# Propofol is the induction agent of choice for urgent intubations with UK physicians

**DOI:** 10.1186/cc9573

**Published:** 2011-03-11

**Authors:** KD Rooney, R Jackson, A Binks, A Jacques, RT IC-Severn

**Affiliations:** 1 Bristol School of Anaesthesia, Bristol, UK

## Introduction

We performed a multicentre, prospective, observational study across nine hospitals in the Severn Deanery (UK). Choice of induction agents for out-of-theatre intubations was compared against historical controls.

## Methods

Data were collected prospectively on all out-of-theatre tracheal intubations occurring within the region during a 1-month period. We included all intubations performed outside areas normally used for elective or emergency surgery. Neonates and cardiac arrests were excluded from analysis. Data were collected locally using a standardised proforma and centrally collated. All intubations were performed according to the preference of the treating team.

## Results

Hypnotics were used for 164 out-of-theatre intubations. Seventy-six per cent of intubations were accomplished by the use of propofol. Propofol was more likely to cause hypotension than other hypnotics (27.4% vs. 14.3%). Use of alternatives increased with seniority of the intubator. Consultants and senior trainees were less likely to use propofol than junior trainees (73% vs. 93%). Etomidate was not used at all. Previous studies from North American and European centres demonstrate greater use of alternative induction agents, particularly etomidate and ketamine [1-4]. UK practice has also changed over time, comparing our study with historical controls [5,6].

## Conclusions

There is significant geographical variation in choice of induction agent for critically ill patients. There has been an increase in the use of propofol amongst UK physicians over the past 7 years. Choice of hypnotic agent has a significant impact on physiological stability and out-of-theatre intubations are commonly performed in emergent circumstances on unstable patients. This study raises concerns that UK physicians choose induction agents based on familiarity rather than the pharmacodynamic profile.
